# A novel modified hanging maneuver in laparoscopic left hemihepatectomy

**DOI:** 10.1016/j.ijscr.2020.10.002

**Published:** 2020-10-08

**Authors:** Kosei Takagi, Yuzo Umeda, Takashi Kuise, Ryuichi Yoshida, Kazuhiro Yoshida, Kazuya Yasui, Yuma Tani, Takahito Yagi, Toshiyoshi Fujiwara

**Affiliations:** Department of Gastroenterological Surgery, Okayama University Graduate School of Medicine, Dentistry, and Pharmaceutical Sciences, Okayama, Japan

**Keywords:** Hanging maneuver, Laparoscopic, Liver resection

## Abstract

**Introduction:**

The liver hanging maneuver is an essential technique for controlling bleeding in hepatectomy, however it is often difficult in laparoscopic major hepatectomy. The present study describes a novel modified hanging maneuver in laparoscopic left hemihepatectomy.

**Presentation of case:**

A 29-year-old female underwent laparoscopic left hemihepatectomy for mucinous cystic neoplasm. After mobilizing the left lobe, the liver parenchyma was dissected along the demarcation line. For the hanging technique, the upper edge of the hanging tape was placed on the lateral side of the left hepatic vein, and fixed with the Falciform ligament. The lower edge of the tape was extracted outside the abdomen. Accordingly the hanging tape can be controlled extraperitoneally during the liver parenchyma dissection.

**Discussion:**

This technique includes several advantages including no need of assistance using forceps, easy control of the hanging tape extraperitoneally, outflow control, better exposure of surgical field, and helpful guide of the liver dissection line toward the root of the left hepatic vein.

**Conclusion:**

Our novel modified hanging maneuver is easy and reproducible to use in laparoscopic left hemihepatectomy. Moreover, this technique can be applied to other laparoscopic hepatectomy.

## Highlights

• The liver hanging maneuver is an essential technique in open hepatectomy.
• Few studies have reported on use of hanging maneuver in laparoscopic hepatectomy.
• We have developed a novel modified laparoscopic hanging maneuver technique.
• This novel modified hanging maneuver is easy and reproducible to use.

## 1 Introduction

The advantages of minimally invasive surgery over open hepatectomy have been shown with lower complication rates and shorter hospital stay [1]. Since laparoscopic hepatectomy has become a standard procedure, the indication of laparoscopic major hepatectomy is internationally increasing [2]. Intraoperative bleeding control during laparoscopic hepatectomy is a major issue to perform procedures safely. Although the liver hanging maneuver is a helpful technique for outflow control mostly used in open hepatectomy, few studies have reported on use of hanging maneuver in laparoscopic left hemihepatectomy [3]. In addition, the hanging maneuver is technically challenging due to the difficulty of extrahepatic dissection and encirclement around the left hepatic vein (LHV). To overcome these problems, a modified liver hanging maneuver, which does not require the dissection of the LHV with the upper end of the hanging tape placed on the lateral side of the LHV, has been reported in laparoscopic left hemihepatectomy [4]. However these conventional hanging maneuver techniques in laparoscopic hepatectomy have still concerns regarding that an assistant has to use forceps to hang up the liver using the hanging tape. Therefore these conventional hanging maneuver techniques could not be performed without assistance by an assistant. To solve these concerns for hanging maneuver in laparoscopic hepatectomy, we have developed a novel modified hanging maneuver technique which does not require assistance. The present study demonstrates our novel modified hanging maneuver in laparoscopic left hepatecomy. The present study is described in accordance with the SCARE Guidelines [5].

## 2 Presentation of case

**Fig. 1:**
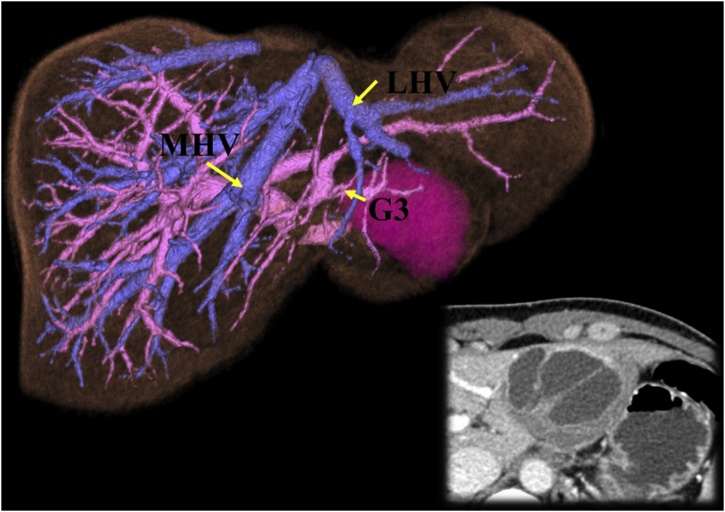
The three-dimensional imaging based on computed tomography showed mucinous cystic neoplasm, located at the segment III in the form of compressing the root of the Glissonean pedicle of segment III. MHV, middle hepatic vein; LHV, left hepatic vein; and G3, Glissonean pedicle of segment III.

A 29-year-old female was referred to our hospital with the suspicion of mucinous cystic neoplasm of the liver. The patient had no previous medical history including drug history, family history, and psychosocial history. The tumor was located at the segment III in the form of compressing the root of the Glissonean pedicle of segment III (Fig. 1). In addition, the computed tomography and magnetic resonance imaging revealed the potential risk of malignancy, therefore we decided to perform laparoscopic left hepatecomy instead of left lateral sectionectomy. Hepatic functional reserve was normal with indocyanine green (ICG) retention rate at 15 min of 5.9% and the Child-Pugh grade A (score 5).

**Fig. 2:**
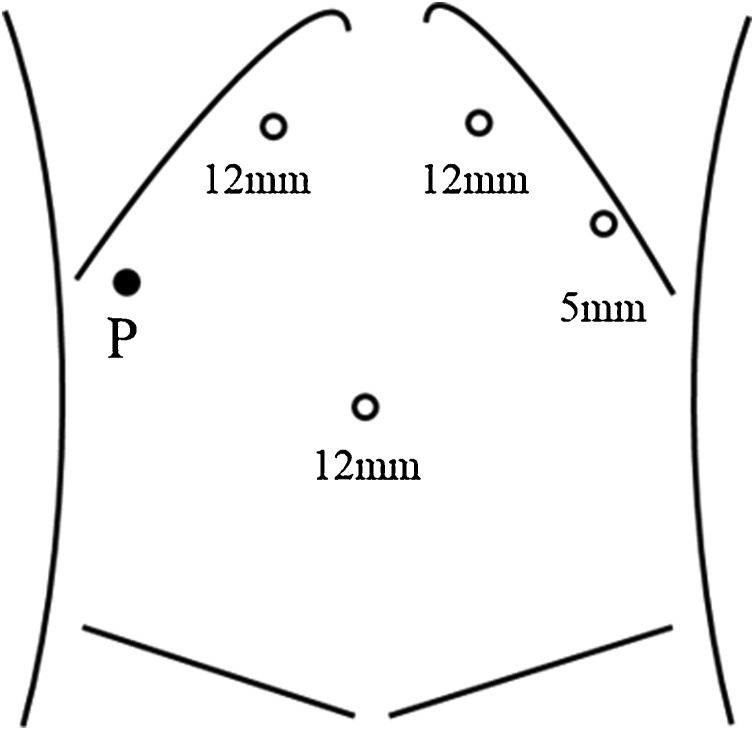
Trocar placement using 4 trocars technique for laparoscopic left hemihepatectomy. P, Pringle’s maneuver.

The patient was placed in the supine position with the operator between the legs and the assistant and scopist at the left side. Four trocars technique was introduced at the umbilical portion for the camera and the both subcostal areas for the operator and assistant (Fig. 2). Firstly the left lateral lobe was mobilized with transection of the Falciform ligament and left triangular ligament. After cutting off the Arantius’s ligament, the left extrahepatic Glissonean pedicle was encircled. Secondly the liver dissection line was marked on the ischemic demarcation line after clamping the left Glissonean pedicle. Intermittent Pringle's maneuver was applied for inflow control using a tourniquet system at the right subcostal area (Fig. 2). The liver parenchyma was dissected using the Cavitron Ultrasonic Surgical Aspirator (CUSA) and Ultrasonic shears (Ligasure).

**Fig. 3:**
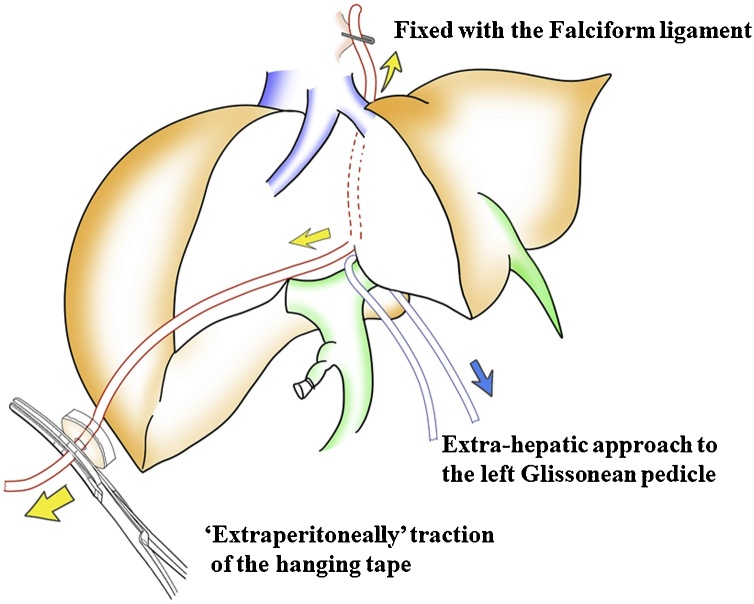
The overview of a novel modified hanging maneuver technique.

**Fig. 4:**
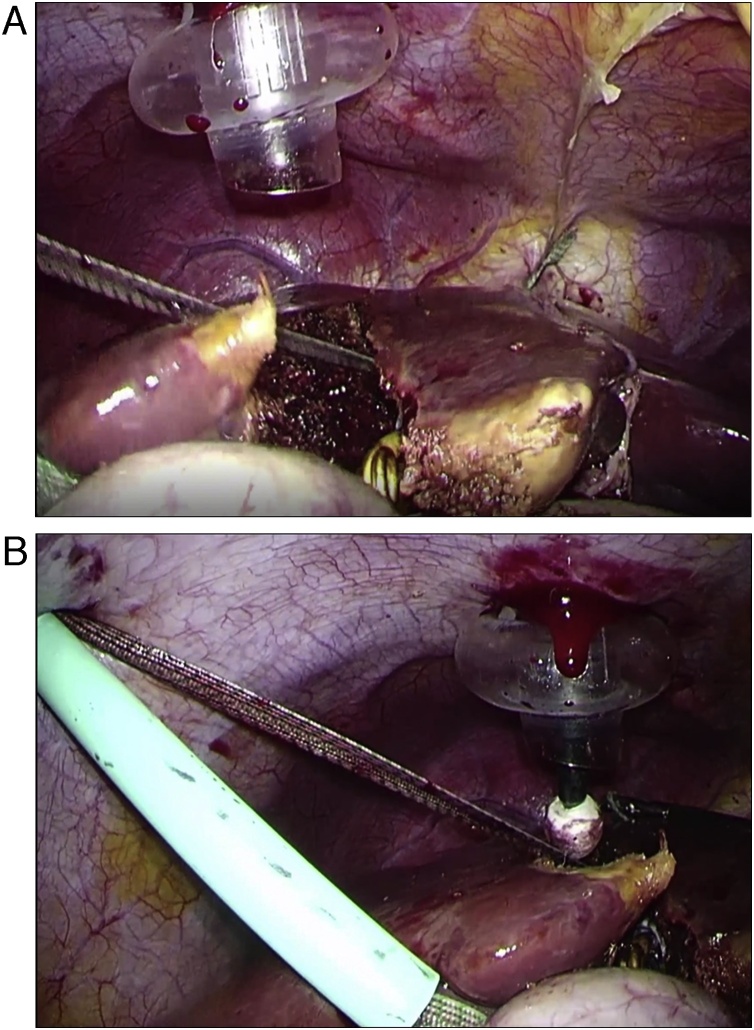
**a** The upper edge of the hanging tape was placed on the lateral side of the LHV, and fixed with the Falciform ligament using clips. **b** The hanging tape was positioned along the left side of the middle hepatic vein, and the lower edge of the hanging tape was extracted along the tourniquet for the Pringle's maneuver.

The overview of our novel modified hanging maneuver is represented in Fig. 3. For this hanging technique, the upper edge of the hanging tape was placed on the lateral side of the LHV, and fixed with the Falciform ligament using clips (Fig. 4a). The hanging tape was positioned along the left side of the middle hepatic vein, and the lower edge of the hanging tape was extracted along the tourniquet for the Pringle's maneuver (Fig. 4b). This hanging technique does not require assistance by an assistant for hanging the tape up. Moreover, the hanging tape can be controlled extraperitoneally. During the liver parenchyma dissection, the hanging tape was pulled out not only to control the bleeding from the outflow, but also to expose a better surgical field. In addition, the hanging tape was used as a guide toward the root of the LHV.

Finally the left Glissonean pedicle and LHV were divided with the stapler, and the specimen was extracted through the Pfannenstiel incision. The total operative time was 280 min, including the Pringle's maneuver for 45 min, and the estimated blood loss was 35 mL.

The patient was discharged on postoperative day 7 without any complications. The final pathological examination of the liver specimen was diagnosed as mucinous cystic neoplasm of the liver without malignancy.

## 3 Discussion

The present study demonstrates a novel modified hanging maneuver in laparoscopic left hemihepatectomy. This technique can solve several concerns of the conventional hanging maneuver in laparoscopic hepatectomy. Moreover, this technique can be applied to other laparoscopic hepatectomy such as left lateral sectionectomy, extended left hemihepatectomy including the middle hepatic vein, and even right-sided hepatectomy.

The biggest concern of the conventional hanging maneuver in laparoscopic hepatectomy would be that the number of trocars is limited for an assistant. Normally five trocars technique was introduced for left hemihepatectomy [2], that means two trocars are available for an assistant. When an assistant uses forceps for the hanging maneuver, other tasks such exposure of dissection line or using a suction might be limited. Furthermore, four trocars technique would be quite difficult for these conventional hanging maneuver [3,4].

Several advantages of this technique should be acknowledged. First, an assistant does not have to lift the hanging tape up using forceps as the tape is fixed with the Falciform ligament. This could wave additional trocars for the conventional hanging maneuver [3,4], that leads to the reduced trocars. Actually we performed the procedure with four trocars technique. Second, the hanging tape can be easily controlled by pulling the lower edge of the tape up or down extraperitoneally. Third, the hanging tape can guide the dissection line of the liver. We mean that the liver dissection along the hanging tape can guide to reach the root of the LHV. Using the guide of the hanging tape could avoid misleading toward the LHV.

The present study includes limitations by the fact that this technique is based on our experience and should be validated in other cases. However we believe this laparoscopic hanging maneuver could be reproducible and used even in other laparoscopic hepatectomy including the left lateral and right lobe resection.

## 4 Conclusion

This novel modified hanging maneuver is easy and reproducible to use in laparoscopic left hepatectomy. In addition, this technique can be applied to other laparoscopic hepatectomy.

## Declaration of Competing Interest

The authors have no conflicts of interest to declare.

## Funding

Not applicable.

## Ethical approval

Because this was a single report, and not a trial or observational research, there was no requirement for ethical approval.

## Consent

Written informed consent was obtained from the patient for publication of this case report and any accompanying images.

## Author contributions

All authors contributed to this work, and approved the final manuscript.

## Registration of research studies

Not applicable.

## Guarantor

Kosei Takagi.

## Provenance and peer review

Not commissioned, externally peer-reviewed.
